# Implementation of an internet-based stress management program in micro- and small-sized enterprises: a study protocol for a pre-post feasibility study of the effectiveness-implementation hybrid type 2 trial

**DOI:** 10.1186/s40814-024-01481-9

**Published:** 2024-04-05

**Authors:** Natsu Sasaki, Taichi Shimazu, Hajime Takeno, Sayaka Ogawa, Utako Sawada, Akizumi Tsutsumi, Kotaro Imamura

**Affiliations:** 1 Department of Mental Health, Graduate School of Medicine, The University of Tokyo, Tokyo, Japan; 2 Division of Behavioral Sciences, National Cancer Center Institute for Cancer Control, National Cancer Center, Tokyo, Japan; 3 Department of Psychiatric Nursing, Graduate School of Medicine, The University of Tokyo, Tokyo, Japan; 4 Department of Public Health, Kitasato University School of Medicine, Sagamihara, Japan; 5 Department of Digital Mental Health, Graduate School of Medicine, The University of Tokyo, 7-3-1, Hongo, Bunkyo-Ku, Tokyo, 113-8655 Japan

**Keywords:** Internet-based intervention, Stress prevention, Occupational health, Implementation science, Health equity, Mixed-methods

## Abstract

**Background:**

Although internet-based stress management programs are proven effective in improving mental health among workers, micro- and small-sized enterprises (MSEs), lacking in occupational healthcare services, face challenges implementing them. To address this gap, this study will develop the program with stakeholders at MSEs to aim for real-world implementation.

**Objectives:**

This paper describes a study protocol for a pre-post feasibility study of an effectiveness-implementation hybrid type 2 trial of text-based internet-based programs (“WellBe-LINE”) in MSEs with less than 50 employees. This feasibility study primarily aims to evaluate trial methods for future effectiveness-implementation hybrid type 2 trials.

**Methods:**

For this study protocol, an internet- and text-based self-care intervention program using the LINE app (a popular message tool in Japan) will be prepared according to evidence-based psychoeducational topics. Based on our online survey findings, personalized algorithms will be implemented according to employees’ gender, age, and psychological distress levels. A personalized program using a popular pre-existing text app is expected to reduce employees’ burdens and be attractive to them, resulting in successful implementation outcomes and mental health benefits. A pre-post design feasibility study will be conducted on ten companies to evaluate trial methods (e.g., recruitment and procedures). The primary outcome will involve individual-level penetration, defined as the proportion of the number of employees who register for the program divided by the total number of invited employees at the company. The progression criterion to go next trial specifies that more than 50% of the recruited companies obtain 60% individual penetration, which is set based on the findings of the prior survey of employees at MSEs and of interviews of stakeholders involved in this study, and will be measured by LINE system. Finally, acceptability, appropriateness, and feasibility will be measured using internet-based questionnaires and interviews.

**Discussion:**

This pre-post feasibility study for future effectiveness-implementation hybrid type 2 trials will provide in-depth knowledge about the successful implementation of text-based, semi-personalized, self-care mental health interventions in real-world settings using both quantitative and qualitative data.

**Conclusions:**

This feasibility study will help validate the effectiveness of text-based interventions using a widely used social networking service (SNS) tool for employees in MSEs.

**Trial registration:**

UMIN clinical trial registration, UMIN000046960. Registered on February 21, 2022.

https://center6.umin.ac.jp/cgi-open-bin/ctr/ctr_view.cgi?recptno=R000053570

**Supplementary Information:**

The online version contains supplementary material available at 10.1186/s40814-024-01481-9.

## Contributions to the literature

- Internet-based mental health interventions in micro- and small-sized enterprises have not been implemented yet.
- This feasibility study plans to test the implementation strategies to achieve high penetration in employees.
- This study can provide insights into occupational health implementation in a disadvantaged context.

## Background

Worldwide, mental health problems in the workplace impact individual health-related disability and productivity loss [1, 2]. Micro-, small-, or medium-sized enterprises (MSMEs) are important targets for mental health interventions [3] as they represent about 90% of businesses and more than 50% of employment worldwide [4]. However, MSMEs, especially micro- and small-sized enterprises (MSEs) with fewer than 50 employees, are less likely to implement health promotion programs because of limited resources, such as cost, access, and time [5]. Thus, offering evidence-based programs alone may not be valuable for employees. While implementing the primary prevention of mental health in MSMEs is challenging [3], preventive interventions for mental health is generally more effective when used by a large population [6]. Therefore, increasing the adoption and penetration (reach) of health promotion program is  the key objective. To achieve high penetration, interventions and strategies specific for MSEs must consider implementation barriers.

Previous studies have identified barriers to the implementation of mental and health interventions in small companies [7–12]: low leadership engagement of employers [7], lack of knowledge about the impact on business [8], limited resources [9, 12], limited time and money [11], regulations (no legal requirement) [12], lack of understanding on the necessity of the interventions, and suboptimal approach [10]. Additionally, job-related stressors between small and large companies varied [13]. In small companies, poor psychosocial factors at work and poor communication are strongly associated with job stress [13]. Therefore, personalized intervention content and specific delivery strategies to overcome organizational barriers in MSEs are needed.

Internet-based interventions are potential options to solve problems of implementing mental health interventions in MSEs, considering high ownership rate of smartphone in Japan. Internet-based intervention is feasible, cost-effective, and accessible [14–16], meeting the needs and addressing the low resource (i.e., limited time and money, lack of human resource and occupational health services) at MSEs. A comparison of face-to-face and internet-based interventions revealed no differences in their effectiveness in treating common mental disorders [17, 18]. Even text message-delivered interventions have been proven to be effective for stress management [19]. Including the occupational health settings, the effectiveness of the internet-based interventions without a face-to-face component has been shown in meta-analysis [15, 16].

Moreover, tailoring (or personalizing) messages in internet-based interventions proves even more effective in stimulating changes in health behavior [20]. Internet-based and personalized text message interventions may thus be suitable for improving the acceptance of employees, leading to successful implementation in MSEs. Low-intensity programs can alleviate the burden on workers, and personalized online programs with ease can effectively address stigma at MSEs. Additionally, programs presented in easily understandable language address a wide variety of employees’ knowledge about mental health, resulting in acceptability. Acceptability, appropriateness, and feasibility have been suggested as important implementation aspects of mental health interventions [21–24]. However, few studies have not been conducted yet examined the effectiveness and implementation outcomes of internet-based interventions for MSEs without some recent protocols [25, 26]. Therefore, methods to improve implementation outcomes (i.e., implementation strategy) should be developed and examined.

As MSME managers have little motivation to implement the program [5, 7], a specific implementation strategy personalized to the context should be adopted. According to the Expert Recommendations for Implementing Change (ERIC), educating stakeholders (employees, employers/managers, and recruiters) is a possible strategy if leadership engagement can be a barrier in the context [27, 28].

Informing employers about the importance of preventive measures in mental health and effective procedures for introducing the program to employees may increase their adoption. However, no study has investigated the effect of internet-based interventions on MSEs with such implementation strategies (i.e., educating employers). Previous implementation research for mental health interventions using the internet or technologies suggested the negative attitude of stakeholders (and, particularly, the users) for internet-based interventions (more preference for face-to-face) [29, 30] and require education for providers [31]

Hybrid-type designed studies can be conducted between effectiveness studies and implementation research [32]. The hybrid type 2 design can be used to test the effectiveness in general practice settings without controlling/ensuring delivery of the intervention and implementation process [32]. Moreover, feasibility and pilot studies [33] can help build and test effective implementation strategies by addressing uncertainties around design and methods and identifying potential causal mechanisms [34]. According to the CONSORT 2010 statement: extension to randomized pilot and feasibility trials, pilot or feasibility studies should have clear criteria for deciding whether to progress to the next full trial [35, 36]. Because there are still many uncertainties in MSEs settings and less evidence is available in implementation research, a pilot feasibility study is important to obtain insights about how we should act in the next stage.

### Aims and objectives

This pre-post feasibility study on a future effectiveness-implementation hybrid type 2 trial aims to evaluate the implementation trial procedures of text-based online programs in micro- and small-sized enterprises with less than 50 employees.

The objectives of this feasibility study, in preparation for a future trial, are as follows:

1. To evaluate the feasibility of trial methods (e.g., recruitment, and procedures) and strategies by setting individual-level penetration as an indicator of successful implementation (primary outcome).
2. To evaluate the barriers and facilitators at MSEs to increase individual-level penetration for revising the strategies.
3. To evaluate the acceptability, appropriateness, and feasibility of the program among recruiters, employers/managers, and users.
4. To evaluate the fidelity and cost of delivering the program to employees among recruiters and employers/managers.
5. To ensure no harm on psychological distress of the users.

## Methods/design

### Program outline of “WellBe-LINE”

**Fig. 1:**
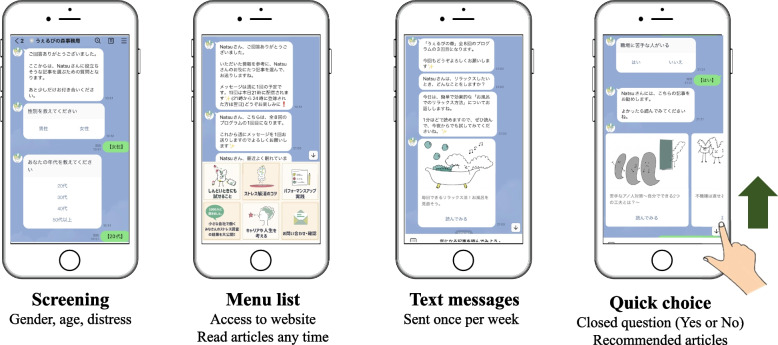
Outlook of “WellBe-LINE”

**Fig. 2:**
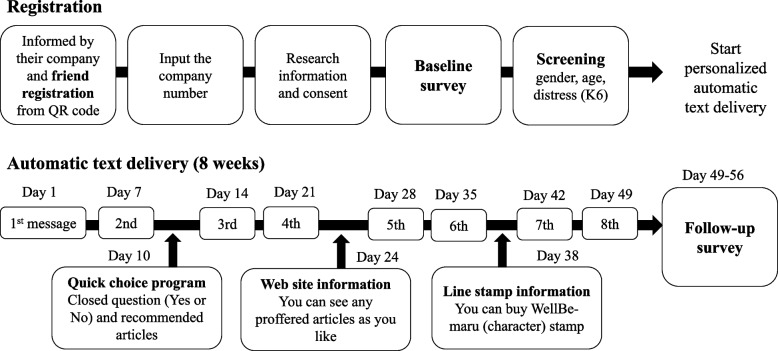
Procedures of starting “WellBe-LINE” and schedule of the intervention

The text-based self-care intervention program “WellBe-LINE,” which is customed for employees in MSEs, will be set in the LINE app (commonly used SNS chat tool in Asian countries) based on evidence-based psychological contents. One text message will be sent once per week and includes a website link (URL) for more information. Figure 1 presents an outlook on the message. The portal website (https://wellbeing-kokoro.com/) contains more than 100 articles on mental health, comprising topics about problem solving [37], acceptance and commitment therapy [38], self-compassion [39], sleep hygiene [40], cognitive behavioral therapy for insomnia [41], and physical activity [42]. Figure 2 presents the procedures for starting the program and the intervention schedules.

### Personalization

A systematic review found that personalization is a key characteristic that promotes attractiveness and acceptability of mobile health interventions [43], through reducing end-users’ acquisition costs. Since lack of awareness of mental health is one of the challenges at MSEs [44], personalization potentially lowers the hurdles to use the program. Personalizing programs are effective at creating changes in health behavior [20], maximizing effectiveness despite the low intensity of the program. We provide 16 scenarios according to the 16 groups (gender × four age categories × psychological distress [K6 ≥ 5, or K6 < 5]) by screening check when participants started the program. Supplementary File 1 presents detailed information about the personalized program. “WellBe-LINE” provides messages in order of rank, from highest to low (week 1 to 8). Supplementary File 2 reports the final personalized scenario for the 16 groups. The efficacy of the “WellBe-LINE” itself has not been examined.

### Study design

In the feasibility study, a one-repeated-measure (pre-post) design will be used. Measurements will be taken at baseline (pre) and 8 weeks afterwards (post). Data collected will then be used to further refine the intervention, recruitment, procedures, strategy for disseminating, and power of a subsequent cluster RCT, which is a hybrid type 2 trial. This study protocol was approved by the Research Ethics Committee of the Graduate School of Medicine, Faculty of Medicine, University of Tokyo (2021190NI-(1)). The trial registration is available elsewhere (UMIN000046960).

### Participants

At the organizational level, companies with fewer than 50 employees will be recruited. At the individual level, employees over 18 years old will be recruited, regardless of their employment contract (e.g., part time). As this study examines the implementation of preventive programs for all employees, no further exclusion criteria are needed. Individuals not using LINE, without intentions to use LINE, or without devices will not be potential participants, while they will be informed about this research.

### Recruitment and procedure

To activate MSEs’ employers/managers, we will use a pre-existing trustful pipe with the MSEs. Five licensed social insurance consultants (recruiters), who are patient public involvement (PPI) members, recruited MSEs that they had a relationship with. Recruiters provide the research information and invitation to demo program (short version of “WellBe-LINE” which finishes in 1 week). If the invited company is interested in participating in the research, the recruiter will provide contact information for researchers. The researchers will then coordinate with the employers/managers on the date of the online meeting (described in the next paragraph).

After the meeting, researchers provide one poster and one original clear file for each employee. Using a template, researchers will also e-mail employees to invite employees to participate in the program. The researcher will provide one “code” for each company. Employers/managers inform employees about the present research by the way they fit the company (e.g., e-mail, posters, and other ways of appealing them). Employees who participate in the program must enter the code after registering for the program via their personal LINE account. Research details will then be explained on the LINE chat, and they will provide their concert by pushing the button to start answering the baseline survey. Employees select their sex and age categories. They also answer six questions about psychological distress (measured by K6). The program automatically recognizes their characteristics (sex × age × distress [5 + high or low]) and starts the program. Employees informed the sensitive information (i.e., the private account information of LINE, psychological distress) will be securely handled by The University of Tokyo under ethical approval and will not be disclosed to the company they are employed through the announcement from the employers/managers and the printed paper material (poster). This study will include individuals with exceeding psychological distress threshold; however, it is justified following two aspects: (i) the program serves as a universal prevention and (ii) high scores of psychological distress are not the same as the psychiatric disorders, instead the reducing the scores is worth in primary preventive interventions.

### Online meeting with employers/managers (implementation strategy)

Licensed social insurance recruiters engage in the recruitment process to motivate relevant employers and managers to participate in the study. After obtaining agreement, researchers will provide 30–45 min of online semi-structured lectures (meetings) with employers and managers via zoom to determine the situation at the company and provide knowledge about behavioral changes and an effective way to invite employees for participation and attain high penetration. A previous qualitative study suggested that fundamental factors influencing the implementation of workplace health promotion in SMSEs entail the leadership engagement of employers [7]. Table 1 presents and justifies the contents of the semi-structured lecture with implementation strategy specification guidelines introduced by Proctor et al. [45] for each component. Semi-structured lectures will then be conducted using Microsoft PowerPoint. Psychological techniques for engaging employees are based on classical behavioral psychology related to motivation theory [46–49] and processing fluency related to health information [50].

**Table 1:** Specification of the implementation strategy per Proctor et al.’s framework (reference [44])

Domain	Strategy 1: Recruitment by licensed social insurance consultants	Strategy 2: Online semi-structured lecture for employers/managers
Actor(s)	Licensed social insurance consultants (cooperators of researchers)	Researchers in the University of Tokyo
Action(s)	Introduce this research to relevant employers/managers of MSEs	Provides online semi-structured lecture via Zoom for 30–45 min
Target of the action	Employers/managers of MSEs agree to participate in this research and to attend the online lecture from researchers in the University of Tokyo	Employers/managers of MSEs announce the intervention “WellBe-LINE” for employees in an effective manner to encourage them to participate as much as possible
Temporality	At licensed social insurance consultants’ most convenient time (e.g., e-mail, poster, and online meeting) Researchers provided tools for licensed social insurance consultants to support the introduction of this study protocol - YouTube (https://www.youtube.com/watch?v=IBEq7ZsWJmc) (https://www.youtube.com/watch?v=E1Y7jU3JO5Q) - Poster - E-mail template	Before announcement in the workplace for employees via Zoom for 30–45 min Contents - Self-introduction and research explanation (5 min) - Importance of mental health measures at MSEs (5 min) - Easy way to improve employees’ mental health (10 min) - Three psychological tips for engaging employees to participate mental health interventions (15 min) - Q and A (5 min) Determining the company’s culture through interview and consulting the strategy to attract employees (5 min)
Dose	No restriction for the number of times to introduce this research	Once before company announcement for 30–45 min
Outcomes affected	Employers/managers’ acceptability, and appropriateness	Employees’ penetration (primary), appropriateness Employers/managers’ acceptability, appropriateness, and fidelity
Justification	Lack of knowledge about the impact of employees’ mental health on business [8] and not understanding the necessity of an appropriate approach [10] prohibits MSEs to adopt the intervention. Familiar licensed social insurance consultants can work as a stakeholder to motivate employers/managers of MSEs to take actions by introducing this research with customed explanations	Fundamental factors that influence the implementation of workplace health promotion in MSEs were the leadership engagement of employers [7]. Educating stakeholders is a possible recommended strategy if leadership engagement can be barriers in the context [27, 28]

*MSEs* micro- and small-sized enterprises

### Measurements

Outcomes will be measured using online self-report questionnaires and interviews with participants (employees), employers/managers, and licensed social insurance consultants (recruiters). Table 2 presents outcomes measured for each stakeholder.

**Table 2:** Quantitative measures, stakeholders, questionnaire assessment points, system detection, and interviews

	Users (employees)		Employers/managers	Licensed social insurance consultants (recruiters)
	Pre (T1)	Post (T2)	Post (T2)	Post (T2)
Implementation outcome of implementation strategies (system detection)				
Penetration (individual level)		✓		
Implementation outcome of implementation strategies (interviews)				
Fidelity			✓	✓
Implementation outcome of implementation strategies (questionnaire)				
Cost			✓	✓
Implementation outcome of the program (questionnaire)				
Acceptability		✓	✓	✓
Appropriateness		✓	✓	✓
Feasibility		✓	✓	✓
Process outcome (system detection)				
Friend block rate		✓		
Process outcome (questionnaire)				
Uncomfortable experience/harms		✓	✓	✓
Adherence		✓		
Health outcome (questionnaire)				
Psychological distress (K6)	✓	✓		
Work performance (HPQ)	✓	✓		
Work Engagement (UWES-3)	✓	✓		
Job satisfaction	✓	✓		
Euthymia	✓	✓		

#### Implementation outcome

- Penetration (primary)

Individual-level penetration is calculated as the proportion of the number of employees who register for the program divided by the total number of employees at the company. The progression criterion [35] is that over 50% of the recruited companies obtain 60% individual-level adoption. The rationale for setting this goal is that from the results of an online survey of 1000 employees at MSEs, we asked the question, “If the company you work for provided you with information about the program registering as a friend on LINE regarding information useful for mental health and work, would you be willing to do so?” Specifically, 44.8% of the respondents answered “Yes or “Fairly agree” to the question. For the second question, “Please select one app or social networking site that you are most likely to use (or most prefer) when receiving mental health or work-related information.” Meanwhile, 35% of the respondents answered “do not want to receive such self-care information.” From these results, it is assumed that the estimated participation ratio would be 45–65% and that 60% of the company’s own employees would be registered as friends on LINE, which would be considered a high level of penetration at the individual level. If we do not achieve the progression criteria, the entire process and delivery of the program will be revised based on the findings of the present feasibility study.

- Fidelity of implementation strategies

The implementation strategy of online semi-structured lecture aims to increase the target action of that employers/managers of MSEs announce the intervention “WellBe-LINE” for employees in an effective manner to encourage them to participate as much as possible (Table 1). To assess the fidelity of this strategy, we will conduct interviews with employers/managers about whether they invite employees following the contents of the lecture (Yes/No).

- Cost of implementation strategies

Cost will be assessed through questionnaires with employers/managers. The time spent on program delivery will be assessed.

- Acceptability, appropriateness, and feasibility of the program

Acceptability, appropriateness, and feasibility will be measured using questionnaires and interviews with employees (users) and employers/managers. Implementation outcome scales for digital mental health (iOSDMH) will be used in a questionnaire to assess three domains of implementation outcomes [24]. The iOSDMH has three versions (i.e., users, providers, managers, and policymakers). The items of the iOSMDH were developed through a literature review, and the outcome was organized according to Proctor’s implementation outcomes [22]. The response options include a 4-point rating for users and 5-point rating for providers and for managers or policy makers, with the added option of “Don’t know.” In this study protocol, employees will then be asked to use iOSDMH for users and employers/managers by iOSDMH for providers. If employers and managers (or people who are concerned and provide the program in the company) are clearly separated, iOSDMH will be used by managers, policymakers, and providers, respectively.

#### Health outcome

- Users’ psychological distress

Psychological distress will be measured using K6 (Kessler 6) [51, 52]. Respondents will be asked to report how frequently they had experienced the following six symptoms in the past 4 weeks: felt nervous, hopeless, restless or fidgety, worthless, depressed, and felt that everything was an effort. Response options include “none of the time,” “a little of the time,” “some of the time,” “most of the time,” and “all of the time. Scores range from 0 to 24. The Japanese version of the K6 exhibits good reliability and validity [53]. K6 works well as the CIDI Short Form in identifying cases of clinically significant mental disorders [52]. Scores over 5 are judged as having moderate psychological distress and can be used as a cutoff point for high or low distress [54, 55]. Reduction of the scores of psychological distress is a commonly used outcome in preventive interventions in the mental health field because it leads to reducing the risk of incidence of psychiatric disease [56].

- Work performance (HPQ)

Work performance will be evaluated using one item of the WHO Health and Work Performance Questionnaire (HPQ) [57]. Participants will then be asked to rate their overall work performance over the past 4 weeks. Items are scored on a 10-point scale ranging from 0 (worst) to 10 (best), with high scores indicating good work performance. The Japanese version of the HPQ exhibits good reliability [58].

- Work engagement (UWES-3)

In the ultra-short form of the Utrecht Work Engagement Scale, three items (UWES-3) will be used to assess work engagement [59]. The UWES-3 consists of three subscales (i.e., vigor, dedication, and absorption) with each of one item. The UWES-3 is a self-reported 7-point rating scale (0 = never; 6 = everyday). The mean score of the three UWES subscales, and total score is computed by adding the scores and dividing the sum by the number of items in each subscale. The Japanese version of the UWES-3 exhibits good reliability and validity [60].

- Job satisfaction

Job satisfaction will be measured using one item from the Brief Job Stress Questionnaire (BJSQ) [61] on a 4-point Likert scale. Higher scores indicated higher job satisfaction.

- Euthymia

Euthymia is a transdiagnostic construct for well-being and represents psychological flexibility, a unifying outlook on life, and resistance to stress [62, 63]. The euthymia scale (ES) is a 10-item index with dichotomous options (False = 0; True = 1). This results in total scores ranging from 0 to 10, indicating better euthymic state for higher scores. The Japanese version of the ES has high concurrent validity and sensitivity as a clinimetric scale [64].

#### Process outcome

- Block rate

LINE accounts can reject or “block” contact from other accounts. We will assess this rate of blocking by system detection.

- Uncomfortable experience/harms

Uncomfortable experience/harm will be assessed using five items from the iOSDMH [24]. Time consumption, mental symptoms, induced dangerous experiences regarding safety, physical symptoms, and excessive pressure on regular learning will be measured for employees (users) in the questionnaire. Employers/managers and recruiters will be asked to rate the following item: “This program does not result in negative side effects (e.g., physical or psychological symptoms).” The response options are 4-point rating scales for users and 5-point rating scales for providers (managers or policy makers), with the added option of “Don’t know.”

- Adherence (completion rate)

Whether employees read or engaged with messages could not be accurately detected in LINE owing to technical limitations. Instead, we asked employees to answer four questions related to adherence in the follow-up survey: “How many times did you read the LINE message?” “How many times did you visit the website?” “Did you visit other websites when you received a notification on LINE?” and “Did you read other related articles in the website?”.

### Analysis

#### Sample size

As this is a feasibility study, a formal sample size calculation is not required [35], but we set the target sample size to maximum of 10 companies (200 employees), giving the variety of characteristics of the MSEs. We discussed with stakeholders, who collaborate the next definitive trial, about that this size is enough to proceed to it and agreed. This study protocol also aims to inform sample size in our future definitive trial, while the effect size estimates of feasibility study can be deviated [65]. This study protocol will support our decision to proceed to the next large trial and will be utilized for our revisions to the program and implementation strategy.

#### The criteria for success of feasibility

This study aims to test a set of feasibility objectives [36] (i.e., trial methods and implementation strategies (online training)). Testing the feasibility is ensured about whether the strategy works for behavioral changes of employers and providers and to improve the targeted primary implementation outcome (penetration). The decision about to proceed the next trial will be made based on the criteria of success of feasibility (i.e., the primary implementation outcome: penetration), which more than half of the participated company achieve 60% of the company’s own employee’s registration to the program. See the section of penetration (primary) in details.

#### Analysis of other quantitative data

Health outcomes will be evaluated using a paired *t*-test between pre-(T1) and post-intervention (T2) according to protocol. The effect sizes and 95% CIs will be calculated using Cohen’s *d* only for those who completed the post-intervention questionnaire. For subgroup analysis, we will conduct the same analysis in the subgroup divided by program completion (completer/non-completer) and satisfaction (satisfied/unsatisfied). Statistical significance will be defined as *P* < 0.05. IBM SPSS Statistics® version 28 will be used for all analyses.

#### Analysis of qualitative data

Semi-structured interviews for stakeholders will be conducted after interventions and will be transcribed at the same time during interviews without audio-recording to make the setting secure to talk about negative matters. The interview data will be used to evaluate the barriers and facilitators to provide the program to employees at their worksite and to evaluate the impact of the implementation strategy (i.e., online meetings with employers/managers) on penetration. These evaluations will be used to develop further strategies in the next trial. To determine the barriers and facilitators, descriptive data from the interview will be summarized and informed by the Consolidated Framework for Implementation Research (CFIR) [66]. One interviewer will categorize the description, and all interviewers will confirm its relevance. The priority of the determinants to be addressed in the next trial will be decided by voting and rating. If it is needed, program adaptation and developing new implementation strategies will be followed. In that case, alternative implementation strategies will be developed referring ERIC [28] and following implementation mapping [67]. We will describe the process and experience of making decisions for future cluster RCT.

#### Patient and public involvement (PPI)

PPI facilitates enhanced quality, appropriateness of research, and development of user-friendly research materials [68]. In our study, seven PPI partners knew about the real context of MSEs: four licensed social insurance consultants, one occupational health physician who provided services in MSEs, one employer in MSEs (one of co-authors, HT), and one manager in the Tokyo Chamber of Commerce and Industry who organized the “Health and Productivity Management.” These PPI partners have participated in all research stages, including the program development, user-relevant research questions, user-friendly materials, more appropriate recruitment strategies for studies, discussion of the interpretation of data, and dissemination of study results. The PPI process is described based on the PPI handbook and reporting checklists [69, 70].

## Discussion

This study protocol paper aims to evaluate implementation outcomes via the “WellBe-LINE” in MSEs with less than 50 employees. “WellBe-LINE” is a text-based, semi-personalized, self-care mental health intervention program through the LINE app. The program sends messages with content considered effective at preventing mental issues [37–42]. This pre-post feasibility study for future effectiveness-implementation hybrid type 2 trials will provide in-depth knowledge about the successful implementation of self-care interventions in real-world settings using both quantitative and qualitative data. Any adverse events and/or unsuccessful procedures will be utilized to identify alternative strategies before proceeding to the next full trial. No digital mental health intervention specifically for MSEs has not been successfully implemented yet. Thus, the present feasibility study should provide new insights to the both field of occupational health and implementation science.

This study protocol will apply the specific implementation strategy wherein researchers meet with employers/managers online for 30–45 min to encourage them to invite employees to participate in the “WellBe-LINE” Program. This strategy is based on previous findings of leadership engagement being a considerable barrier in the health program implementation [7]. Furthermore, PPI partners who engaged in all research stages will provide essential suggestions in implementation, leading to a positive impact on field-specific studies [68].

### Limitation

This study protocol has several limitations. First, the sample size may not be adequate to detect significant effectiveness in reducing psychological distress owing to the pilot nature of this study. Second, this study protocol lacks a control group; the true impact of the implementation strategy can only be determined in the next step. Third, generalizability of the study findings is limited owing to its challenges in the recruitment process. As with the definitive trial, only the MSEs which have high interests in health promotion can be the potential participants through the current recruitment process. Future studies could engage and recruit MSEs with low interests in health promotion may be required in studies with another research focus. Fourth, individual-level penetration may be affected by personal beliefs about mental health issues in the workplace (e.g., social desirability [71], fear of stigmatizations [72], concerns about negative impact at the workplace [73]). Thus, factors not related to the implementation strategy can be considered if the outcomes are evaluated. Fifth, digital interventions risk marginalizing vulnerable groups, such as people with difficulty using devices or without internet access. Ensuring the opportunity to provide the program for all employees is essential in future consideration. Sixth, we will not provide any additional supports to participants with high psychological distress since it is justified under the aim of primary prevention and the contents are relevant to reduce distress, but there still be a potential risk to make conditions worse. Seventh, this feasibility study will collect many exploratory items from participants to utilize the findings in future implementation research. Still, it may cause participants’ burden, resulting in the response bias. Although authors limit the items to as few, the potential burden should be acknowledged. Eighth, fidelity of implementation strategies is evaluated using one simple question, instead of using systematic approach, such as a checklist. Other actions of employers/managers to be achieved through the online lecture should be defined after this feasibility trial to develop a checklist.

## Conclusion

MSEs are less likely to implement health promotion programs worldwide, although many employees work there. The low-cost and easy-to-access self-care program “WellBe-LINE” targeted specifically at the needs of MSEs can be a solution, despite its low intensity, if the penetration of employees is applicable. The wide range of evaluations proposed in this study protocol will provide valuable suggestions for implementing preventive health promotion measures in MSEs. Additionally, the study findings can be potentially applied to the program concept and strategies in similarly disadvantaged and marginalized settings, resulting in the promotion of health equity.

### Supplementary Information

**Additional file 1. **Supplementary files 1 and 2.

## Data Availability

The data related to the present study protocol are available from the corresponding author, KI, upon reasonable request.

## Abbreviations

BJSQ: Brief Job Stress Questionnaire
MSEs: Micro-, small-sized enterprises
MSMEs: Micro-, small-, or medium-sized enterprises
PPI: Patient and public involvement
